# Saunders’ Question Compends

**Published:** 1903-03

**Authors:** 


Saunders' Question Compends.—Essentials of Diseases of the Ear.—By E. B. Gleason, S. B., M. D., Clinical Professor of Otology, Medico-Chirurgical College, Philadelphia; Surgeon in Charge of the Nose, Throat, and Ear Department of the Northern Dispensary, Philadelphia, etc. Third edition, thoroughly revised. 16 mo. volume of 214 pages, with 114 illustrations. Philadelphia and London: W. B. Saunders & Co., 1902. Cloth, $1.00, net.

In this valuable little aid the diagnosis and treatment of diseases of the ear has been handled briefly yet clearly and concisely and without sacrificing accuracy of description. Students and those practitioners who are contemplating taking a post-graduate course in otology will find it well worth a careful reading. The price being merely nominal, this, as is true of the rest of Saunders' Question Compend Series, will command for it a large sale. R.

